# COVID-19 pandemic and antimicrobial resistance in developing countries

**DOI:** 10.15190/d.2021.6

**Published:** 2021-06-30

**Authors:** Abdul Rehman Arshad, Farhat Ijaz, Mishal Shan Siddiqui, Saad Khalid, Abeer Fatima, Rana Khurram Aftab

**Affiliations:** ^1^CMH Lahore Medical College and Institute of Dentistry, Abdur Rehman Road, Cantt, Lahore, Pakistan; ^2^Dow Medical College, Dow University of Health Sciences, Mission Road, New Labour Colony Nankwara, Karachi, Pakistan; ^3^Punjab Institute of Cardiology, Lahore, Pakistan

**Keywords:** COVID-19, antimicrobial resistance, developing countries, antibiotic resistant bacteria.

## Abstract

A wide range of antimicrobial agents were touted as potential remedies during the COVID-19 pandemic. While both developed and developing countries have recorded an increase in the use of antimicrobial drugs, use and misuse have occurred to a far greater degree in developing countries. This can have deleterious consequences on antimicrobial resistance, especially when various developing countries have already reported the emergence of various drug-resistant organisms even before the pandemic. Telemedicine services, societal and cultural pressures, and bacterial co-infections can predispose to overwhelming antimicrobial prescriptions. The emergence of new multidrug resistance species is a major concern for the developing world especially since health services are already overburdened and lack the diagnostic capabilities and basic amenities for infection prevention and control. This can lead to outbreaks and the rampant spread of such microorganisms. Improper waste management and disposal from hospitals and communities establish freshwater runoffs as hubs of various microorganisms that can predispose to the rise of multidrug-resistant species. Microplastics' ability to act as vectors for antibiotic-resistant organisms is also particularly concerning for lower-middle-income countries. In this review, we aim to study the impact of antimicrobial use during the COVID-19 pandemic and antimicrobial resistance in lower middle-income countries, by understanding various determinants of resistance unique to the developing world and exploring solutions to combat the problem.

## SUMMARY

*1. Introduction*

*2. Determinants of Antimicrobial Resistance in the Developing World*

*3. Antimicrobial Self-Prescription*

*4. Restriction of Health-Care Services*

*5. Social and Cultural Reasons*

*6. Antibiotic Overprescription in Hospitals and Telemedicine*

*7. In Hospital Emergence of Antimicrobial Resistance*

*8. Environmental Factors: Role of Freshwater, Sewerage and Plastic Waste*

*9. Conclusion*

## 1. Introduction

As of April 21, 2021, the Coronavirus disease 2019 (COVID-19) has affected more than 143 million people and has caused 3 million deaths worldwide^1^. The causative organism of COVID-19, the Severe Acute Respiratory Syndrome Coronavirus 2 (SARS-CoV-2), is an enveloped, positive-sense single-stranded RNA virus. Various medications have been touted as a remedy for the disease, ranging from homemade concoctions to antimicrobial drugs with potentially severe side effects, such as hydroxychloroquine. Various unsubstantiated claims of several of these medications' efficacy resulted in immediate rampant misuse of such antimicrobial agents among both hospitals and the general population. This has raised concerns for increasing antimicrobial resistance. Whilst extensive literature exists on the impact of antimicrobial use during the COVID-19 pandemic in developed countries, little to no literature is available on lower middle-income countries, where an already fragile antimicrobial regulation infrastructure exists and governing of the availability and selling of antimicrobials is scant. In this review, we aim to study the impact of antimicrobial use during the COVID-19 pandemic and antimicrobial resistance in such countries by understanding various determinants of resistance unique to developing countries. Moreover, we aim to explore possible solutions to combat the problem.

A more justified use of antimicrobials in COVID-19 patients is to treat potential superinfections. Risk factors that can add to the burden of bacterial disease include a dysregulated immune response, lowered host defenses, and prolonged in-hospital stay^2^. The treatment of such superinfections is pertinent to improve COVID-19 prognosis, as secondary bacterial infections have been linked to increased morbidity and mortality^3^. Such rampant use of antimicrobial agents raises concerns for misuse, that can translate into antimicrobial resistance and the rise of multi-drug resistant organisms that have already been anticipated to be the world's leading cause of death by the year 2050^4^. For example, prophylactic azithromycin may have a possible role in transmitting extensive drug-resistant strains of Salmonella typhi in Pakistan and Bangladesh^5^. It has already been established that in-hospital transmission of multi-drug resistant organisms further increases the likelihood of resistance development^6^.

There are also several contributory factors arising from the general population that promote the development of antimicrobial resistance in the setting of COVID-19 in developing countries. These include inappropriate prescriptions, lack of antimicrobial stewardship practices, and unnecessary self-administration by the public^3,7-9^. Developing countries also suffer from inadequate hygiene in communities and hospitals, financial constraints, over-the-counter availability of antibiotics, and misinformation on various media, all of which have been implicated as risk factors for antimicrobial resistance^10,11^.To our knowledge, our study is the first study that discusses the impact of antimicrobial use during the COVID-19 pandemic on the emergence of antimicrobial resistance in developing countries.

## 2. Determinants of Antimicrobial Resistance in the Developing World

**Figure 1:**
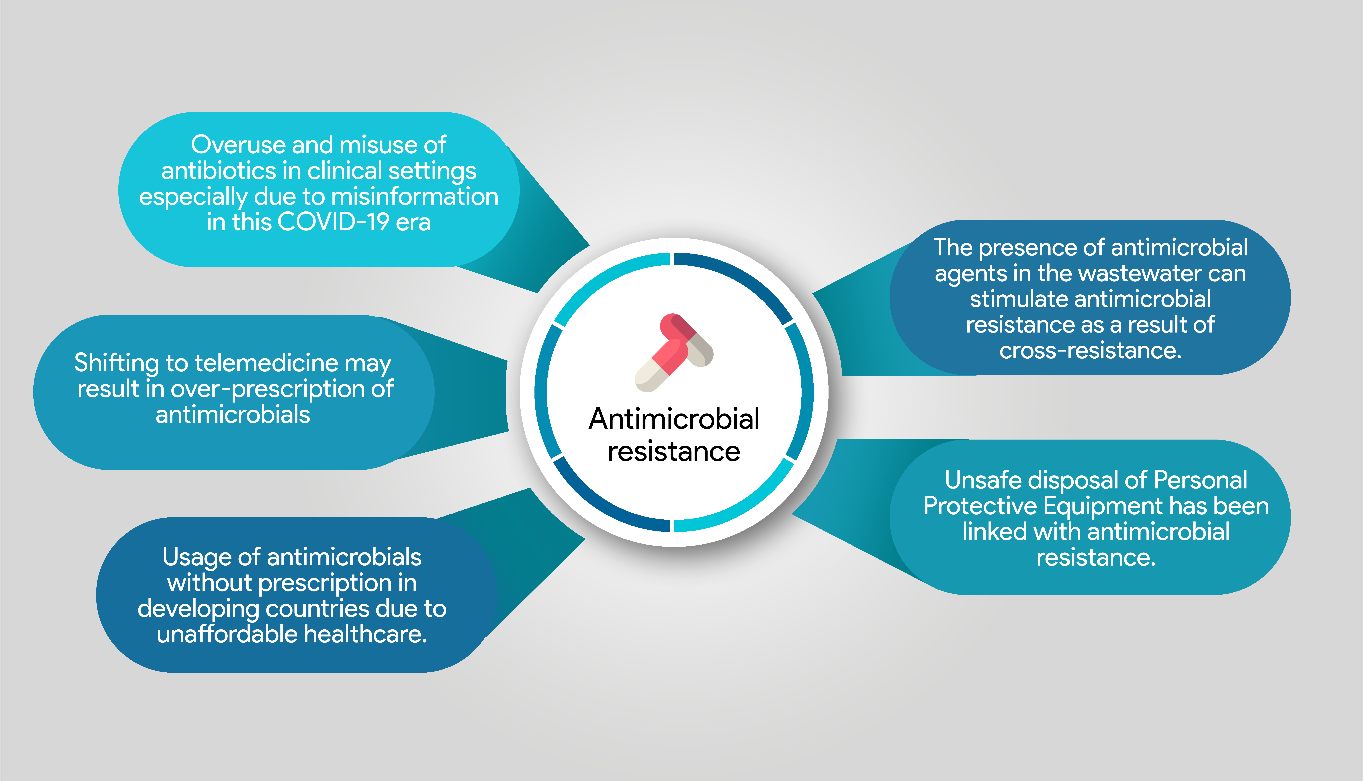
Determinants of antimicrobial resistance in the developing countries in the aftermath of COVID-19

The development of antimicrobial resistance in the setting of coronavirus pandemic is attributed to numerous factors (Figure 1). The trend of self-medication due to unaffordability and insufficiency of healthcare services compounded by misinformation and unregulated content on social and broadcast media can be ascribed to overuse of antimicrobial agents for management of COVID-19.

Amidst the COVID-19 pandemic, where general public was instructed to maintain social distancing, the utilization of telemedicine also surged. Although convenient for both the patients and physicians, the lack of physical examination by healthcare professionals and unavailability of laboratory parameters for determining the severity of the condition are one of the major causes of excessive prescription of antibiotics.

Unsafe disposal of personal protective equipment serves as a possible cause of emergence of antimicrobial resistance. The microplastics derived from healthcare waste act as media for propagation and modifications in bacteria and thereby consequent emergence of resistant species.

Due to expenses and techniques associated with standard disposal of personal protective equipment, the waste produced in healthcare setup is usually disposed in sewerage water. The abundance of various antiseptics and other miscellaneous antimicrobial agents in the wastewater can cause various modifications in bacteria secondary to cross-resistance with various substances present in sewerage wastewater. This cross-resistance can possibly result in emergence of more resistant species.

## 3. Antimicrobial Self-Prescription

During the course of the COVID-19 pandemic, it has been observed in several developing countries that there was a general aversion towards seeking healthcare from hospitals and an inclination towards self-medication and pharmacies^12,13^. Appreciable increase in the use of antibiotics was observed in Pakistan^13^, and Bangladesh^14^, and a vast majority of such use was without the prescription of a health professional^14^. The most frequently prescribed medications included ivermectin, azithromycin, and doxycycline^14^, which are key drugs utilized as the mainstay of COVID-19 treatment at the time of the conduction of studies^15^. This was hypothesized to be abetted by the relative stability in the prices of these medications in developing countries^13^, which is also supported by the findings of Fernandez et al. who state that the growing unstable financial conditions render more appropriate therapy and supervised medications as unaffordable to several families in developing countries and persuade families to otherwise use readily available and comparatively cheaper antibiotics^16^. Stemming from the mental stress of lockdown and isolation, there was a general fear of shortage of antibiotics too, that further exacerbated widespread antimicrobial and antibiotics use^14^, which indeed did cause a shortage of medications as observed in India, Pakistan, Bangladesh, and Malaysia^14^.

The problems of self-prescription can be solved by widespread educational programs that spread awareness about the disease and address the mass hysteria and misconceptions that follow such a pandemic.

## 4. Restriction of Health-Care Services

The burden of antimicrobial resistance during a pandemic has already been suggested to differ for different world regions^17^. Lower middle-income countries were predicted to be the pallbearers and experience the greatest toll due to poorer health care standards, poor antibiotic prescription habits, and greater population densities^17^. Other generalized factors included the ability of the microbe to spread readily. With strict lockdowns in several regions of the world, this notion was initially accepted. However, in-depth mathematical models proved differently^18,19^. Strict lockdown measures could cause a temporary 50% reduction in tuberculosis transmission, but principally, due to the accumulation of undetected tuberculosis during this period, there could be an estimated 1.19 million cases and 361,000 deaths in India, 24,700 cases and 12,500 deaths in Kenya, and 4,350 cases and 1,340 deaths in Ukraine in the next five years^18^. The most significant overall impact of the lockdown on tuberculosis mortality and cases in regions with high antibiotic resistance was the drop in completion of the second-line treatment^18^. Delayed first presentation of tuberculosis cases most likely due to social stigma surrounding it, as it can present very similar to COVID-19, has been voiced as a major concern^18,20-22^. This creates a reservoir of undetected tuberculosis that can precipitate new infections of latent tuberculosis^18,19^, which has been suggested to lead to selection for drug resistance^6^, whilst deficient tuberculosis management programs can lead to increases in the rates of multi drug resistant tuberculosis (MDR-TB) observed globally^23^.

## 5. **S**ocial and Cultural Reasons

Hernando-Aman et al. argue that lower middle-income countries are more prone to acquire antimicrobial resistant bacteria due to socioeconomic or cultural reasons^24^. Sociocultural aspects such as uncertainty avoidance, inequalities, gender-bias, and individuals' integration into primary groups highly influence antibiotic use and antibiotic resistance. They argue that it is intrinsic human behavior to avoid being excluded from a higher social group, and deviation of common social rules is often considered a mental disorder in lower middle-income countries. Thus, along with a lack of education on antibiotic resistance and antimicrobial resistant bacteria, people in lower middle-income countries are generally encouraged to partake in antibiotic misuse^24^.

## 6. Antibiotic Overprescription in Hospitals and Telemedicine

The ideal time to start antibiotics in COVID-19 cases still represents a grey area in clinical guidelines. According to the guidelines laid down by the World Health Organization (WHO), antibiotics should not be administered in uncomplicated cases of COVID-19. They should only be reserved for more severe, complicated cases^25^. Studies, however, demonstrate a prudent divergence from these guidelines. In one study, antibiotics were prescribed in 72% of COVID-19 cases on the clinical suspicion of a bacterial co-infection. However, only 7% of these patients actually developed bacterial and/ or fungal co-infections, demonstrating the overwhelming unjudicial misuse of antibiotics^26^. It can also be expected that such statistics may indeed be much higher in the developing world, owing to a multitude of predisposing factors. However, it is not to say that we can completely determine the unjustified use of antibiotics. A lack of controlled longitudinal studies on this particular subject makes it difficult to ascertain if such antimicrobial use was truly unjustified or if it served a protective prophylactic role in reducing the incidence of co-infections.

Antimicrobial stewardship has been introduced to effectively utilize antibiotics, curtail their misuse, and minimize antimicrobial resistance risk. However, poor adherence to antimicrobial stewardship has been reported in several developing countries, and a lack of literature on the subject has made it difficult to understand the cause^10^. Thus, it is necessary that antimicrobial stewardship principles are strictly adhered to and supervised by regulatory bodies during the COVID-19 pandemic.

In lieu of social distancing protocols during the COVID-19 pandemic, telemedicine has been a major preference to clinical visits. This has been noted to impact routine clinical examinations of patients significantly^27^. One study describes the significant over-prescription of antibiotics in telemedicine visits as compared to routine clinical visits^27^. Immune system dysregulation in the setting of COVID-19 has also been noted to alter the clinical manifestations of an underlying bacterial infection^28^. Thus, the difficulty in accurately assessing a patient requires the physician to extend beyond his/her clinical acumen and utilize laboratory testing to investigate the infection as bacterial in nature. We propose the development of inexpensive, sensitive testing methods that provide rapid diagnosis of bacterial infections. We also propose that doctors in developing countries be educated and encouraged to prescribe antibiotics only according to antimicrobial stewardship principles and WHO guidelines.

## 7. In Hospital Emergence of Antimicrobial Resistance

While the use of antibiotics in certain patients’ populations can be debated to be justified or unjustified, there exists a large subset of COVID-19 patients in whom the use of antimicrobial agents is crucial. These include those patients in whom a higher incidence of bacterial infections has been observed, notably by multi-drug resistant microorganisms. It has already been established that in-hospital transmission of multi-drug resistant organisms increases the likelihood of development of antimicrobial resistance^5^.

Due to the ever-evolving nature of microorganisms and their interdependence with humans, the environment, and animals, it is not uncommon for diseases, such as COVID-19, Ebola virus disease, and Zika virus, to spread from their zoonotic origin to humans and to cause epidemics and pandemics^29,30^. By undergoing changes in their genetic material and bypassing active drug pathways, several microorganisms have acquired antimicrobial resistance^30^. It has also been noted that antimicrobial resistance can induce re-emergence of past microbes as well as new resistant variants that possess increased pathogenicity^30^.

### 7.1 Candida Species

A part of COVID-19 patients develops acute respiratory distress syndrome (ARDS), along with potential super-infections^3^. Because *Candida *species are a significant constituent of the normal human flora, they have been implicated in opportunistic infections in COVID-19 patients^31^. Nosocomial *Candida *species were noted to be particularly detrimental even before the COVID-19 pandemic. Thus, various guidelines were devised to minimize its transmission and develop new antifungal agents^32,33^.

During the COVID-19 pandemic, various yeast infections have been elaborated on, but the emergence of a new multi-drug resistant *Candida *species has been implicated in causing COVID-19 associated candidiasis^31^. Such an emergence of a new *Candida *species has been very concerning, especially since antimicrobial resistance is already ubiquitous^31^. The mechanism by which *Candida *causes co-infections in COVID-19 patients is unknown. However, both *Candida *and SARS-CoV-2 have been found to occur on the same hospital surfaces^32^, and COVID-19 patients hospitalized in the ICU share the same risk factors, medications, and underlying co-morbidities with *C. Auris^32^*. It has been reported that the fecal fungal microbiome is altered in COVID-19 patients with superimposed fungal infections; this has been noted to increase the transmissibility of *Candida *infections amongst COVID-19 patients and can even cause outbreaks in hospitals^33^.

The incidence of the multi-drug resistant *C. Auris *has already been noted to be greater in developing countries^32^. Chowdhary A. et al. postulate this to be due to the overall greater burden of *C. Auris *in developing countries as a result of poor diagnostic capabilities and inadequate resources for infection prevention and control^32^. By utilizing all their limited diagnostic capabilities on SARS-CoV-2, overburdened medical facilities in developing countries cannot attend to the diagnosis of other pathogens, such as *C. Auris^32^*. Hence, the issue of multi-drug resistant *C. Auris *and potential outbreaks are a much greater concern in developing countries than in developed countries^32^.

### 7.2 New Delhi Metallo-Beta-Lactamase-Producing Carbapenem-Resistant Enterobacterales (NDM-CRE)

One of the already established potential causative agents of healthcare-acquired infections in many countries, including developed countries like Italy, is *New Delhi Metallo-Beta-Lactamase-Producing Carbapenem-Resistant Enterobacterales *(NDM-CRE)^34^. *Enterobacterales *species are resistant to the majority of first-line therapeutic agents^35^. COVID-19 patients, who were previously colonized with NDM-CRE and those who acquired it during the course of hospital stay, were found to have a longer hospital stay attributed to associated complications and antimicrobial resistance^34^.

### 7.3 Carbapenemase-producing Enterobacterales Carbapenemase-producing enterobacterales (CPE)

CPE has re-emerged in the light of the COVID-19 pandemic, attributing to inattentive antibiotic prescription and a lack of antimicrobial stewardship^36^.

The re-emergence of CPE and superinfection in COVID-19 patients has multiple underlying factors. The most common factor is the use of immunomodulators, such as corticosteroids, in the management of COVID-19 patients^36^. Other factors include severe lung injury and subsequent mechanical ventilation^36^.

### 7.4 Carbapenem-Resistant Acinetobacter baumannii

There has been reported to be an increased prevalence of *Carbapenem-Resistant Acinetobacter Baumannii *(CRAB) during the COVID-19 pandemic^37,38^. Nosocomial *Acinetobacter Baumannii *infections has been found to be associated with increased mortality in COVID-19 patients^39^. These species have been noted to be resistant to both ciprofloxacin and gentamicin, thereby leading to increased morbidity and mortality in COVID-19 patients^35^.

During the COVID-19 global pandemic, antimicrobial resistance will have long-lasting effects due to increasing resistance, redirection of resources, re-emergence of resistant strains of various infectious agents, and lack of antimicrobial stewardship^40,41^. It has already been expected that lower middle-income countries are likely to suffer from antimicrobial resistance’s consequences in the post-pandemic era to a much greater degree than developed countries^42^. Hence, we propose that the use of antibiotics should not decrease, but rather be used more efficiently, centralizing on preventing in-hospital transmission of multi-drug resistant organisms. This can be achieved by observing strict infection control protocol and patient isolation, a scenario that is often below par in the developing world.

## 8. Environmental Factors: Role of Freshwater, Sewerage and Plastic Waste

Various studies have reported wastewater to be major hotspots for antimicrobial resistance^43-47^. They have been found to be hubs of subtherapeutic antibiotics and very high bacterial loads,^47^and have also recently been found to contain excessive other miscellaneous antimicrobial products such as antibacterial soaps and antiseptics^44,45,47,^. The presence of antimicrobial agents in the environment can stimulate antimicrobial resistance^24,43-46^, and cause cross-resistance between antiseptic agents and antimicrobial agents by inducing mutation in the receptors and pathways which cause antimicrobial resistance, leading to the emergence of multidrug-resistant species^16,44,45^. This explains the emergence of drug-resistant E. Coli collected from sewage treatment plants that were treated using chlorination treatment^46^.

Using the WHO’s strategy for removal of SARS-CoV-2 using sodium hypochlorite disinfection, was found that there was only incomplete removal^48^. This explains the presence and persistence of SARS-CoV-2 in wastewater, sanitation systems, as well as in the septic tanks of hospitals^43^. The authors alarm the readers of how much higher concentration and persistence can thus be expected in developing countries^43^. Only one study has attempted to document the extent of SARS-CoV-2 found in sewage wastewater in a lower middle-income country. It was discovered that there were far more extensive levels of SARS-CoV-2 found that did not correlate with the low number of cases in Quito.

The unsafe disposal of plastic personal protective equipment in the environment too has been voiced as a major concern for antibiotic resistance and antimicrobial resistant bacteria. Cultivable antimicrobial resistant bacteria found on the surface of microplastics were found to be 100-5000 times higher than those in water samples, implying the efficient ability of microplastics to act as vectors for antimicrobial resistant bacteria, particularly superbugs^49,50^. Superbugs on microplastics have been suggested to enrich the antibiotic-resistant bacteria found on the surfaces of other microplastics, and since developing countries lack the infrastructure and financial capability to dispose of microplastics using modern incineration techniques safely, such microplastics are usually disposed of in wastewater, leading to a cascade of events that culminate in antimicrobial resistance^46^.

## 9. Conclusion

The extensive use of antimicrobial agents during the COVID-19 pandemic has created a serious risk of antimicrobial resistance. Developing countries have already been reporting high levels of resistance even prior to the pandemic. Several contributory factors that are unique to developing countries have gained major traction during the COVID-19 pandemic. Unregulated prescription of antibiotics to the general public, unjudicial use of antibiotics in health care settings for co-infections, negation of the principles of antimicrobial stewardship, the emergence of multidrug resistant microorganisms, freshwater disposal of toxic waste, and inappropriate management of microplastics are all contributing factors towards the emergence of resistance in developing countries. This necessitates emergency intervention to prevent further deterioration of the situation.

## KEY POINTS

**◊** *Superinfection can occur in COVID-19 patients secondary to dysregulated immune response, weakened defense mechanisms and prolonged hospital stay*

**◊** *Most common factors contributing to the antimicrobial resistance during the COVID-19 pandemic are: self-administration of antibiotics, telemedicine, lack of antibacterial stewardship, over-prescription of antibiotic drugs, unsafe hospital waste management and disposal.*

**◊** *Timely interventions, such as implementation of antimicrobial stewardship, proper waste management, and easily accessible healthcare facilities are imperative to counter evolving antimicrobial resistance in developing countries.*
